# Are vaccination programmes delivered by lay health workers cost-effective? A systematic review

**DOI:** 10.1186/1478-4491-7-81

**Published:** 2009-11-03

**Authors:** Adrijana Corluka, Damian G Walker, Simon Lewin, Claire Glenton, Inger B Scheel

**Affiliations:** 1 Health Systems Program, Department of International Health, Bloomberg School of Public Health, Johns Hopkins University, 615 N Wolfe Street, Baltimore MD 21205, USA; 2 Preventive and International Health Care Unit, Norwegian Knowledge Centre for the Health Services, Oslo, Norway; 3 Health Systems Research Unit, Medical Research Council of South Africa, South Africa; 4 Department of Global Health and Welfare, SINTEF Technology and Society, Oslo, Norway

## Abstract

**Background:**

A recently updated Cochrane systematic review on the effects of lay or community health workers (LHWs) in primary and community health care concluded that LHW interventions could lead to promising benefits in the promotion of childhood vaccination uptake. However, understanding of the costs and cost-effectiveness of involving LHWs in vaccination programmes remains poor. This paper reviews the costs and cost-effectiveness of vaccination programme interventions involving LHWs.

**Methods:**

Articles were retrieved if the title, keywords or abstract included terms related to 'lay health workers', 'vaccination' and 'economics'. Reference lists of studies assessed for inclusion were also searched and attempts were made to contact authors of all studies included in the Cochrane review. Studies were included after assessing eligibility of the full-text article. The included studies were then reviewed against a set of background and technical characteristics.

**Results:**

Of the 2616 records identified, only three studies fully met the inclusion criteria, while an additional 11 were retained as they included some cost data. Methodologically, the studies were strong but did not adequately address affordability and sustainability and were also highly heterogeneous in terms of settings and LHW outcomes, limiting their comparability. There were insufficient data to allow any conclusions to be drawn regarding the cost-effectiveness of LHW interventions to promote vaccination uptake. Studies focused largely on health outcomes and did illustrate to some extent how the institutional characteristics of communities, such as governance and sources of financial support, influence sustainability.

**Conclusion:**

The included studies suggest that conventional economic evaluations, particularly cost-effectiveness analyses, generally focus too narrowly on health outcomes, especially in the context of vaccination promotion and delivery at the primary health care level by LHWs. Further studies on the costs and cost-effectiveness of vaccination programmes involving LHWs should be conducted, and these studies should adopt a broader and more holistic approach.

## Background

In 1978, the Alma-Ata Conference put forward the goal of 'Health for all by the year 2000' and declared primary health care (PHC) the vehicle through which this goal was to be achieved [1]. As a result, PHC service delivery programmes using community or lay health workers (LHWs), a cadre of health worker that was often comprised of ordinary people with minimal health training, were established in many low- and middle-income countries (LMICs) and also became more widespread in high-income settings [2]. However, a combination of factors throughout the developing world in the 1980s, such as economic recession, political and policy changes, population growth, poor governance, and inadequate health systems, led to reduced investments in primary health care, including in LHW programmes [2,3]. Today, a key challenge of health systems in many countries is the need to develop and strengthen human resources to deliver essential interventions [4,5]. This has been a key factor in rekindling interest in the use of LHWs [6,7].

In 2005 Lewin et al. [8] published a Cochrane systematic review examining the global evidence from randomized controlled trials (RCTs) on the effects of LHWs programmes, as compared to usual primary and community health care. This review indicated promising benefits, in comparison with usual care, for LHW interventions in the areas of vaccine promotion; breastfeeding promotion and treatment for selected infectious diseases. However, these results were based only on a limited number of studies. For example, the review identified only three RCTs examining the effectiveness of LHW programmes in improving vaccination uptake. An update of the original review by Lewin et al. [8] to identify and synthesize the results of more recent studies on LHW programmes is being undertaken. An interim report on the updated review identified six trials of vaccination promotion by LHWs [9].

With its focus on RCTs of effectiveness, the original review [8] did not explore factors influencing the costs and cost-effectiveness of LHWs in delivering health services such as vaccinations. Taking intervention costs and effectiveness considerations into account is important for policy decisions and concerns around the affordability of resource inputs for health worker programmes. For governments and funding agencies, the question of whether an intervention is more or less cost-effective compared to alternative interventions, as well as whether there are sufficient funds to pay for the intervention, are factors that influence decision-making. Part of the growing interest in LHW programmes is related to the perception that they are cheaper than those that use professional health staff. However, a health programme is defined as affordable only if each individual or organization financially contributing to the programme is willing and able to contribute to financing its operation on the scale envisioned in the programme design [10]. A greater problem in health programming, from the perspective of those funding these initiatives, is the widespread failure to analyze the future recurrent cost implications of a proposed investment programme and to assess whether these costs will be affordable given available financing sources [10].

These considerations have practical implications for economic evaluations of health worker programmes, and specifically LHW programmes. Generally, conventional economic evaluations, particularly cost-effectiveness analysis, focus narrowly on health outcomes, and do not take into account the role of human-made institutions in shaping economic behaviour. Nor do current economic evaluation methods capture social non-health benefits, such as community empowerment and higher social capital, which may have positive or negative values, and are related to programme-induced changes in the wider community [2]. Through their overly reductionist perspective, conventional economic evaluations of LHW programmes are ill-equipped to deal with institutional changes [11], such as changes in local governance or differences in social values, which are especially important at the community-level. Institutional economics, alternatively, considers the social norms and networks which govern individual and group behaviour and are an important dimension to consider when looking at the cost-effectiveness of LHW programmes. For example, the training of programme staff and other activities that are seen as institution-building, with benefit flows beyond the duration of the programme, are treated as a resource input when valuating outcomes. However, within an institutional economics framework, they may also be considered an intermediate output, with its entire cost subject to amortization as per capital costs [11].

Two non-systematic reviews have indicated the general dearth of cost-effectiveness data on LHW programmes [2,12]. Similarly, three systematic reviews focussing on LMICs, one on the effects and costs of expanding immunisation strategies [13], the other a systematic review of the grey literature on strategies for increasing coverage of routine immunisations [14], and the third a review of published and grey literature on routine immunisation [15], demonstrated the paucity of cost-effectiveness data on strategies to expand the coverage of vaccination services in developing countries. What continues to be missing, however, is a targeted review of the costs and cost-effectiveness of involving LHWs in vaccination programmes. As part of a wider study on LHW programmes for vaccination uptake in low- and middle-income countries (LAYVAC), a systematic review of the costs and cost-effectiveness of using LHWs to promote or deliver vaccinations was conducted.

The overall aim of this paper was to review the costs and cost-effectiveness of vaccination programme interventions involving LHWs. This paper sought to:

1. Identify studies which evaluate the costs and cost-effectiveness of vaccination programme interventions involving LHWs;

2. Summarize included studies narratively and evaluate them according to a methodological quality checklist;

3. Identify factors that contribute to the costs and cost-effectiveness of LHWs and vaccine interventions, and examine how theories of institutional economics can contribute to understanding the costs and cost-effectiveness of LHW programmes.

## Methods of the review

### Selection criteria

This study used Lewin et al.'s [8] definition of a LHW as any health worker carrying out functions related to health care delivery; trained in some way in the context of the intervention, usually informally and related to the job; and having no formal professional or paraprofessional certificate or degree-conferring tertiary education. The term 'LHW' is thus necessarily broad in scope and includes providers involved in both paid and voluntary care. For this review, any type of LHW (paid or voluntary) was included, such as community health workers, village health workers, cancer supporters, birth attendants and medical auxiliaries. Studies on vaccination programmes, be they linked to health promotion activities, vaccine delivery, etc., for both children and adults were included. Full economic evaluations were defined according to Drummond et al.'s [16] definition as 'the comparative analysis of alternative courses of action in terms of both their costs and consequences.' No economic evaluation designs were excluded. Studies involving LHWs and vaccination programmes and including any costing information were included for secondary analysis of LHW activities and costs. Studies in languages other than English, Spanish or French were excluded.

### Search strategy for study identification

The following electronic databases were searched: NHS EED Cochrane Library (Issue 1 2008); NHS-EED Center for Reviews and Dissemination (to February 2009); MEDLINE (1950-February 2009); CINAHL (1982-December 2007); EMBASE (1980 to February 2009); ISI Web of Science (1975 to February 2009); EconLIT (1969 to February 2008); Health Economic Evaluation Database (HEED) (to February 2008); LILACS (Latin American and Caribbean Health Sciences Literature) (to January 2008); African Index Medicus (AIM) (to February 2008); Western Pacific Region Index Medicus (WPRIM) (to February 2008); Index Medicus EMRO (Eastern Mediterranean) (to February 2008); SSRN (Social Science Research Network -- Economic Research Network) (to February 2008).

### Search criteria

Full text copies of all articles that were identified as potentially relevant by either reviewer were retrieved. Each full paper was assessed independently for inclusion by at least two reviewers. When reviewers disagreed the decision was referred to a third reviewer.

The searches included a combination of vaccination, LHW and economic terms. Additional file 1 provides the full details of the search strategy for Medline. Details of strategies for the other databases are available from the authors on request. Reference lists of studies assessed for inclusion were also searched. Reviews by Walker and Jan [2] and Pegurri et al. [13] were used to identify potential studies for inclusion; monographs, technical reports and books were excluded as this review focused on published articles. The authors of all studies included in the update of the Cochrane review by Lewin et al. [8] were contacted to ask whether they had collected costs or conducted cost-effectiveness analyses alongside their study. Authors of studies that met initial screening criteria and where further clarification was needed were also contacted. Studies were included after screening of the full-text article.

### Review criteria

The papers were reviewed using a series of questions based on Pegurri et al. [13], which were adapted slightly to reflect some important aspects of working with LHWs, e.g. level of training, remuneration, sustainability, etc. The review questions were split into two parts: background characteristics and technical aspects (Appendix 1). The aim of these questions was twofold: first, to establish the basis for a descriptive analysis of published evidence and second, to enable a structured evaluation of the studies.

## Results

**Figure 1:**
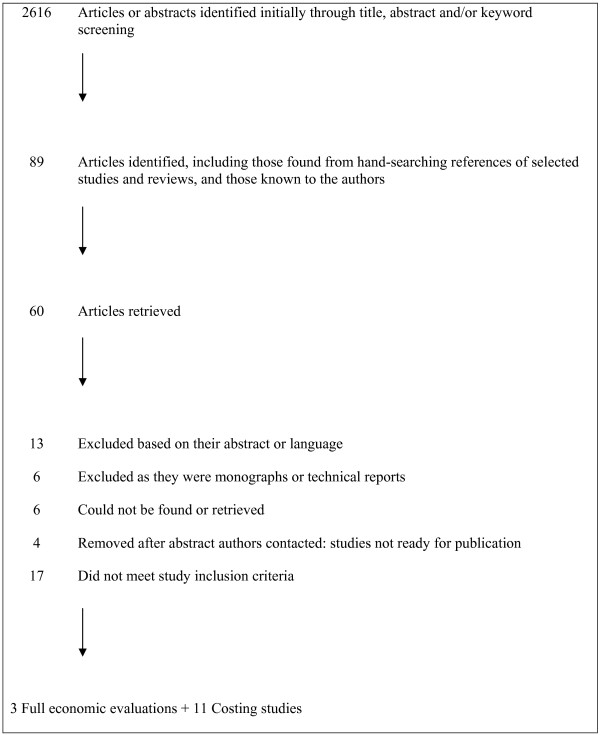
**QUORUM flow chart**.

There were 2616 records identified. Eighty-four of these studies were considered potentially eligible for inclusion and full text articles were then retrieved. Five additional studies were known to the authors or identified from hand-searching references of key studies and reviews once the full-text articles were retrieved, giving a total of 89 articles. Three studies fully met the inclusion criteria of an economic evaluation of a vaccination programme involving LHWs, while an additional 11 were retained as they included some cost data associated with a vaccination programme involving LHWs. Four authors were contacted for papers on the basis of their conference abstracts; however, the papers were not available for inclusion in this study. All included studies were published in English or Spanish language journals. The results of the search are shown in Figure 1 (QUORUM flow chart).

Given the small number of full economic evaluations identified, the following section provides a short description of each. All costs were reported in US dollars (except where noted) and are reproduced here as originally stated in the respective studies (see Table 1 and Additional file 2).

**Table 1:** Background characteristics of the full economic evaluations

	**Deuson et al**. [17]	**San Sebastian et al**. [18]	**Weaver et al**. [19]
Area studied	Philadelphia, USA	Low-Napo area in Napo province, covering 300 km of the Napo river	Seattle, USA
Timing of the study	October 1994 - February 1996	1993-1995	October- November 1996
Type of intervention	Promotion prior to a catch-up campaign^1^	Campaign	Promotion
Type of LHW/role of LHW	Staff of community-based organisation	CHWs*	Senior volunteers, i.e. older people
Training	Unstated	3-year training in preventive medicine, including immunisation, and curative activities	Received training about the pneumococcal and influenza vaccines and received technical support from the project coordinator.
Comparator(s)	(Implicitly) Doing nothing	Centrally planned strategy (District Hospital strategy) of immunizing children <1 year	(Implicitly) Doing nothing
Study type	CEA & CUA	CEA	CEA
Vaccines delivered	Hepatitis B	Routine childhood vaccines	Pneumococcal and influenza vaccines
Age group(s) targeted	2-13 year-olds	0-5 years-old	65 years +
Perspective(s)	Societal	Societal	Societal
$ per child vaccinated	Costs per child, per dose, and per completed series were $64, $119, and $537, respectively	$32 per FVC	Not stated
CE results	The cost per discounted year of life saved was $11,525 and the benefit-cost ratio was 4.44:1	CHW intervention dominated the District Hospital comparison	Intervention cost $35,486/QALY gained for the combined outreach initiative, $53,547/QALY for the pneumococcal vaccine and $130,908/QALY for the influenza vaccine. For seniors who had never received a vaccine, the combined outreach initiative cost $11,771/QALY gained, $38,030/QALY for the pneumococcal vaccine, and $22,431/QALY for the influenza vaccine.
Funded by	Centers for Disease Control (CDC), USA	Medicus Mundi Andalucia, Spain	CDC

* Local indigenous organization started a PHC programme in 25 communities with training of CHWs. Each community has two CHWs with 3 year training in preventive medicine, including immunisation and curative activities. CHWs are literate and elected by their own community and receive no financial reward.

^1 ^Catch-up campaign: targeted efforts to vaccinate individuals that did not receive the vaccine that they would otherwise have received through routine immunisation

Campaign: targeted efforts of vaccinating a group of and/or a pre-determined number of individuals for vaccination

Deuson et al. [17] assessed the value for money of a community-based Hepatitis B vaccination catch-up project for 4384 Asian American children in Philadelphia, USA, implicitly compared with usual care. Staff in the community-based organizations acted as LHWs through educating parents about the hepatitis B vaccination and visited homes of children due for a vaccine dose. Costs per child, per dose, and per completed series were $64, $119, and $537, respectively while the cost per discounted year of life saved was $11 525.

San Sebastian et al. [18] compared the costs and outcomes of two different vaccination strategies for children under five years of age between 1993 and 1995. The District Hospital (DH) strategy was centrally planned and managed by the DH and fully vaccinated five children, resulting in a cost of $777.60 per vaccinated child. The community health worker (CHW) strategy was planned and implemented in conjunction with the CHW Association and fully vaccinated 113 children at a cost of $32 per child.

Weaver et al. [19] conducted an economic evaluation of a community-based outreach initiative to promote pneumococcal and influenza vaccines for people aged over 65 years, compared with no outreach. The authors found that the cost per quality-adjusted life year (QALY) gained was $35 486 for the combined outreach initiative, $53 547 per QALY for the pneumococcal vaccine and $130 908 per QALY for the influenza vaccine. The cost-effectiveness ratio of the intervention targeted to people who had never received the influenza vaccine the previous year was $11 771 per QALY.

The remaining studies did not fulfil the definition of a full economic evaluation but contained some data on the vaccination- and human resource-related costs of vaccination programmes. Of these, four studies looked at LHWs delivering vaccinations only [20-23], five studies evaluated LHWs to promote vaccinations [24-28] (including canvassing, publicizing and persuading people to get vaccinated), and two studies reported using LHWs for both promotion and vaccination [29,30]. Comparing costs in any meaningful way was difficult due to the differences in outcome reporting. More in-depth descriptions of these studies can be found in Additional file 3.

### Background characteristics of the included studies

The included cost-effectiveness studies were diverse in terms of the contexts in which they were conducted and the roles of the LHWs in these settings (see Table 1). The settings of the included cost-effectiveness studies ranged from urban centres in the United States of America, such as Philadelphia [17] and Seattle [19], to sparsely populated communities living along the Ecuadorian jungle river system [18]. LHW vaccination activities included the promotion of Hepatitis B vaccine uptake [17]; routine immunisation [18] amongst children; and the promotion of pneumococcal and influenza vaccination amongst individuals over the age of 65 [19].

The settings of the studies that included some cost data related to vaccination programmes were also very diverse. Of these 11 studies, 10 took place in low- and middle-income countries (Bangladesh [22], Brazil [24], Egypt [30], Haiti [21], India [26], Indonesia [23], Mexico [29], Mozambique [25], Pakistan [20]), and also in West Bank and Gaza Strip [28], while the remaining study discussed the role and costs associated with immunisation registries and follow-up reminders by LHWs for full vaccination coverage in the United States of America [27].

This review also shows highly disparate uses of LHWs (Table 1 and Additional file 2). This ranges from the community-level health worker, with very basic training in delivering preventive health services such as vaccinations at the household level [18,28,30] or outdoor markets [21], to the use of volunteers to promote vaccination uptake amongst those over 65 years of age [19] or door-to-door [25]. Overall, the LHWs in the included studies were used mainly to link communities to vaccination delivery through promotion or campaigns.

Governance issues and institutional characteristics emerged as important factors in determining LHW roles. For example, San Sebastian et al. [18] noted that in the Amazon district of Low-Napo, where their LHW intervention strategy took place, an outreach strategy is required to reach the indigenous population living scattered along rivers, where immunisation coverage is especially low. Compared to the centrally-planned and district hospital implemented vaccination program strategy, the strategy that was planned and implemented with local LHWs was far more effective and successful. LHWs residing in the area are trained to vaccinate as part of their commitment to a PHC programme, and provide nearly half of all out-patient care in the Napo river area. However, their efforts and labour are not always recognized by policy officials [18], which are part of the more formalised institutional and governance structure. In Mexico, researchers found that there were cost-savings when community vaccinators with basic nurse training were used to vaccinate, as compared to the usual delivery of care [29]. They attribute this to factors such as having the same vaccinators within their geographic area of responsibility; constant interaction without conflict between the vaccinator and the community; and allowing the vaccinators the freedom to choose the day and time for home visits.

Recognizing where LHWs can add value in delivering healthcare services, and clearly defining LHW roles and responsibilities is important. In their study in the West Bank and Gaza Strip, Tulchinsky et al. [28] suggest that the village health worker as an all-purpose health provider may be difficult to supervise and sustain. Others have noted that using village health workers for a more selective set of services may be more feasible and manageable when trying to achieve specific targets in disease control [31]. This calls to mind the decades-long debate surrounding 'comprehensive primary health care' versus 'selective primary health care.' Whereas 'comprehensive primary health care' is concerned with a developmental process by which people improve both their lives and life-styles, 'selective primary health care' is concerned with medical interventions aimed at improving the health status of the most individuals at the lowest cost [32]. Narrower or more selective primary health care interventions are easier to evaluate from a conventional economic perspective but, as noted above, such approaches may fail to capture the wider social and institutional changes that may follow these programmes.

### Methodological characteristics

The methodological quality of the included three full economic evaluation studies was good (see Table 2). The viewpoint was explicitly stated by Deuson et al. [17] and could be inferred in the others, with a societal perspective being taken in each case. That is to say, the analyses included all benefits and costs of the programme regardless of who received or paid them, respectively. All important and relevant inputs were identified and valued, with data sources clearly identified. All three studies included economic costs and reported results of sensitivity analyses. Though authors compared their studies to previously published research in order to contextualize their findings, this was insufficient to provide any useful basis for generalizing their findings across time and space.

**Table 2:** Quality checklist (Yes/No/Not Clear/Not stated/Not applicable)

		**Deuson et al**. [17]	**San Sebastian et al**. [18]	**Weaver et al**. [19]
1	Was the viewpoint explicitly stated?	No, but could be inferred	No, but could be inferred	Yes
2	Were all the important and relevant inputs identified and valued given the viewpoint?	Yes	Yes	Yes
3	Were sources of data clearly identified?	Yes	Not stated	Yes
4	Were the unit costs of inputs and quantity clearly identified?	No	Yes	No
5	Was it clear how costs were valued?	Yes	Yes	Yes
6	Is there an attempt to calculate economic costs?	Yes	Yes	Yes
7	Were base year, details about currency conversion and any adjustment for inflation given?	Yes	Yes to base year and currency conversion. No indication of adjustment for inflation.	No
8	Was discounting performed?	Yes	No	Yes
9	If yes, was an appropriate justification of the rate given?	Yes	NA	Yes
10	Was sensitivity analysis performed?	Yes	Yes	Yes
11	If yes, were justifications for the choice of variable and their level given?	Yes	No	Yes
12	Were issues of affordability and/or sustainability discussed?	No	No	No
13	Was generalizability discussed by the authors?	Yes, but not sufficiently	Yes, but not sufficiently	Yes, but not sufficiently

There were fundamental differences in these three studies in terms of:

• variations in context, including differences in setting and location (Philadelphia [17] versus Amazonian Ecuador [18] versus Seattle [19]);

• comparator used (doing nothing [17,19] versus a second strategy [18]);

• intervention design (costs-effectiveness analysis of an education and outreach programme for Hepatitis B vaccination [17], cost-effectiveness analysis of two routine childhood vaccination programmes [18], and a cost-effectiveness analysis conducted alongside a randomized, controlled trial of a community-based outreach initiative [19]);

• outcomes measured (costs per child receiving any dose, per dose delivered, per completed series, and per additional child rendered sero-protected [17]; cost per fully vaccinated child [18] and costs per total QALYs lost because of vaccine side effects, morbidity, and mortality [19]);

• and study populations (Asian American children aged 2--13 years [17]; children aged 0--5 years [18]; and seniors aged 65 and older [19]).

There were some similarities in the times that were costed, but also significant differences between studies in the items that were included. In addition, the same items were costed differently across the three studies, mainly based on their intervention and context-specificity.

• Direct costs: All three studies included vaccine supply costs; however, while Deuson et al. and San Sebastian valued volunteer salaries at unskilled wage rates, Weaver et al. calculated hourly volunteer time by the mean weekly earnings of people aged 65 years and over, divided by 40 (based on a 40-hour work week). Deuson et al. and Weaver et al. included computerized tracking system costs, managing side effects, and hospitalization, and San Sebastian also counted fuel and maintenance costs and per diem allowances. The cost items continued to diverge, as Deuson et al. included inpatient, outpatient, scanner, and laboratory costs for acute and chronic HBV infection, and Weaver et al. included volunteer training costs.

• Indirect costs: the time spent by caregivers on vaccination and travelling, as well as volunteer LHW transportation time, were included and valued at the unskilled wage rate (San Sebastian, Weaver et al.), while medical visits and loss of earnings due to illness were accounted for by Deuson et al.

• Excluded costs: capital costs (land, buildings, shared equipment and administration) and other costs common to the intervention and the comparator were excluded by all studies.

Both the comparability of the findings of these studies and their wider generalizability is hindered by these factors. We address this point in greater detail in the discussion.

Worryingly, issues of vaccination programme affordability and sustainability were largely ignored, though one study [17], noting the increasing administration of vaccines by the private sector, explored the impact of using private sector prices in delivering the intervention. In this study, only the cost of the vaccine, which comprised 8.7% of the total cost of the programme, was varied and other costs, such as community education, outreach and planning, were not [17]. Sustainability issues are discussed in greater detail below.

## Discussion

Despite keeping the inclusion criteria broad and general for sensitivity purposes, and despite systematically searching a large number of databases, there was a dearth of published economic evaluations of LHWs in vaccination programmes. Recently published studies point to the potential expansion of LHW involvement in vaccine delivery, especially related to the latest vaccine-related technological innovations, such as thermostable vaccines [33] and Uniject devices [23]. Combined with the emerging trend of adding more services to immunisation campaigns (e.g. vitamin A, insecticide-treated nets, etc.), we may see more studies reporting the use of LHWs in the future.

The results of the three economic evaluations included in the systematic review show that LHWs were more cost-effective options than the comparator, which did not include LHWs. However, given the diversity in the population groups targeted, as well as in the types of interventions and settings, it is difficult to draw generalizations from these studies. For example, Weaver et al. [19] found that targeting interventions to people who had never received the pneumococcal vaccine or who had not received the influenza vaccine in the previous year improved cost-effectiveness, while Deuson et al. [17] focused on increasing coverage of Hepatitis B vaccination for first-generation children of Asian and Pacific Islander descent, aged between two and 13 years.

The inclusion criteria for this review excluded studies not mentioning lay health workers, vaccines or economic evaluations, or terms related to these. Studies were included when they specifically mentioned LHW involvement in vaccination alongside other health services and indicated costs [26,28]. However, we may have excluded a body of economic evaluation literature concerning the delivery of vaccinations in which LHWs were involved, but packaged with other targeted health services such as family planning interventions. Simmons et al. [34], for example, evaluated the cost-effectiveness of family planning research programmes delivered by LHWs in rural Bangladesh as compared to government programmes; they indicated that vaccines comprised 0.12% of the total programme budget from 1978-1985.

Vaccine delivery by LHWs can be characterized as a complex intervention, whose components usually include behaviours, parameters of behaviours (e.g. frequency, timing) and methods of organizing and delivering those behaviours (e.g. type(s) of practitioner, setting and location); the number of groups or organizational levels targeted by the intervention; and the number and variability of outcomes [35]. To add to the complexity, vaccination programmes are bundled increasingly with other health campaigns, offering a challenge in determining the cost-effectiveness of the immunisation component. For example, a recent cost-effectiveness analysis was conducted of insecticide-treated net (ITN) distribution as part of the 2004 measles vaccination campaign in Togo, with shared costs assumed to be equally attributed between the two health interventions [36]. The results suggested that substantial efficiency gains may be derived from the joint delivery of vaccination campaigns and malaria interventions [36]. Because it is rare for vaccinations or other health services to be delivered in isolation from one another, it is often difficult to determine the indirect costs associated with immunisations in particular. As can be seen by the paucity of full economic evaluations of LHWs and vaccination found in this review, it is also difficult to evaluate the costs associated solely with LHW involvement, mainly due to the interaction of various types of health personnel in service provision. For example, an evaluation of house-to-house versus fixed-site oral polio vaccine delivery strategies in a mass immunisation campaign in Egypt included the costs of physicians, nurses, hygienists, clerks and drivers, in addition to community workers, with differences in personnel costs not only linked to fixed-site versus house-visit, but also linked to urban versus rural areas [30]. Therefore it is difficult, if not impossible, to tease out the contribution of the LHWs.

Like effectiveness outcomes, the costs of (complex) interventions can be strongly determined by contextual factors; by the exact combination and 'dose' of intervention components; or by the behavioural predispositions of participants or providers. A population's attitude toward health care and interventions, compliance and adherence, utility valuations of health status, and incentives---such as level of co-payment---are also important components that can have a significant impact on cost-effectiveness [37]. The difficulty in generalising or transferring economic evaluation results to other settings arises because we do not know what caused the particular relationship between opportunity costs and outcomes in each instance. As interventions become more complex, it becomes even more difficult to explain how a specific bundle of intervention components (and their associated resource use), provided in a given context, has generated the levels and types of outcomes measured [38,39].

### LHWs and institutional economics

The presence of LHWs, and the sustainability of their efforts, also relate to the institutional characteristics of a community. Institutions and institutional characteristics are here defined as the 'rules that govern the conduct of individuals, groups and organizations' [11] and, related to this, the 'patterns of behaviour that determine how individuals, groups and organizations interact with one another' [40]. Institutional economics addresses the role of human-made institutions in shaping economic behaviour, with the understanding that economic analyses and understanding should also consider the political and social system within which economics is embedded.

One of the included studies provides an example of these processes: ongoing demand for the Village Health Room programme between 1985 and 1996 in the West Bank and Gaza Strip overcame political conflict and strains on the delivery of public services, due to both strong community support from the communities served by village health guides and positive recognition by Palestinian health authorities [28]. And, as was noted by San Sebastian et al. [18], involving communities in the planning and implementation of vaccine delivery in the sparsely populated Low-Napo area in Ecuador using the CHW strategy, rather than a top-down district hospital strategy, created community ownership and accountability of the programme, and maximized the cost-effectiveness of immunisation. However, in these cases, conventional economic evaluations failed to capture the 'instrumental value' [11] of LHWs to the community, such as the changes in community norms that may encourage the initiation of further activities and the provision of further services. Furthermore, economic evaluations did not take into account the potential reduction in transaction costs resulting from the LHW being a recognized member of the community, which in itself provides social capital and reduces the amount of time required, as well as the need, to develop new social networks, trust and access to community's resources.

Another example where conventional economic evaluations fail to capture wider, context-specific characteristics is the issue of volunteerism. Within the context of LHWs and vaccine delivery in this review, for example, we found that two studies depended on volunteers for vaccine promotion and uptake [18,19] while the other studies paid the LHWs. The programme intervention of Weaver et al. [19] used a paid programme coordinator, but their strategy also depended heavily on unpaid volunteers. Volunteer labour and paid labour are often used interchangeably, under the assumption that shadow prices for volunteer labour can be substituted for market wages, such as unskilled wage rates [2,16], and the assumption that volunteer and paid staff are equally productive [41]. However, volunteerism, like other forms of labour, is often determined by a different set of personal and social characteristics, and may not be broadly socially patterned or systematic [42]. Furthermore, a community that produces a supply of individuals willing to volunteer may be different to one that does not [2].

Economic evaluations can incorporate such institutional factors by taking a more holistic approach that captures the contribution of health services to the wider community through paying attention to wider community characteristics and impacts. This involves understanding ongoing changes in the ways in which individuals, groups and organizations relate to one another and the full extent of downstream transaction costs [43]. A strong component of the underlying argument for the Alma Ata declaration on primary health care (PHC) thirty years ago, and its emphasis on strengthening health care delivery within a wider definition of health, was that health sector interventions, such as using LHWs for vaccine delivery, can effect institutional changes. As PHC reflects and evolves from the economic conditions, socio-cultural and political characteristics of a country and its communities, and is based on the application of the relevant results of social, biomedical and health services research and public health experience [44], it is critical for economic evaluations of PHC-related activities to include an institutionalist component.

### Sustainability of LHW programmes

Tied to these institutional factors are issues surrounding the sustainability of LHW programmes. Sustainability refers to the continuing ability of a project to meet the needs of its community [45], beyond the period of an intervention [46]. When assessing sustainability, it is useful to differentiate between the sustainability of measured effects, which is difficult to assess when programmes are evaluated for only a few months; the sustainability of the programme's interventions, regardless of its effects (our focus here); and continued financial viability, which is linked to the programme sustainability. Gruen et al. [47] propose that sustainable health programmes be regarded as complex systems that encompass the programmes themselves, the health problems targeted by these programmes and the programmes' drivers or key stakeholders, all of which interact dynamically within any given context. In their systematic review of studies associated with health-programme sustainability, they identified a wide range of factors, including context and resource availability, amongst others [47]. Shediac-Rizkallah and Bone [48] and Bossert [49] note that factors that affect sustainability include programme design, organizational aspects, and contextual attributes including local health policy and social, cultural, and environmental characteristics. As programme sustainability is strengthened by input and support from all facets of the community, this may be linked to the costs that the community and country can afford to maintain, the stage of their economic development, and the importance of community self-reliance and self-determination [50].

The full economic evaluations identified in this review evaluated programmes over a period of two months [19], 18 months [17,19,27] and two years [18]. The costing studies were evaluated over an average of nearly four years (range: nine months [29] to 11 years [28]). Furthermore, in the full economic evaluations, the LHWs were evaluated as part of vaccination promotions or a vaccination campaign, as compared to usual delivery of care, and thus could be perceived as not necessarily being embedded within the health system.

The one study which addressed the issue of sustainability had the longest lifespan of all of the studies, operating for over a decade in the West Bank and Gaza Strip [28]. In this project, the LHWs were young, local women with 10-11 years education, who underwent 6-8 months of training and were paid stipends for their work. They had high levels of prestige in the village and were recognized as an integral part of a health system, as well as being closely supported and supervised by the health system. Issues of sustainability were explored through recognizing the importance of funding, political and administrative support and especially continuity among the guides and supervisory personnel during various transition periods of the programme -- from external funding, to inclusion within the Government Health Services after initial funding ended and in the transition between Israeli and Palestinian administrations. Expansion efforts in 1994 were credited to strong community support for the programme in the villages served and its recognition by Palestinian health authorities.

As this review illustrates, the data available in most cost and cost-effectiveness studies of LHW programmes for vaccination do not allow any rigorous assessment of effect sustainability, programme sustainability or financial sustainability. While these aspects are often difficult to assess within a research framework, given time and resource limitations, they are typically of great interest to decision makers. Researchers therefore need to pay greater attention to assessing the sustainability of the interventions studied and to developing robust methods for evaluating this.

## Conclusion

In his review 'Systematic reviews of economic evaluations: utility or futility?', Anderson argues that it has become increasingly recognised in public health and health promotion that only asking whether an intervention "is effective" has limited value, because effectiveness is more complex and contingent on the specific combination of elements in an intervention, and/or its interaction with different community and organisational contexts [51]. Rather, he argues, it makes much more sense to ask "how and why" an intervention is or is not effective or cost-effective in different circumstances. As noted by Drummond, "there is widespread recognition amongst economists, and possibly amongst decision makers, that whether or not a particular intervention is cost-effective depends on the local situation" [16]. However, a common characteristic of economic evaluation studies in healthcare is that though sensitivity analyses are undertaken to deal with uncertainties in the models, few studies look explicitly at variability between locations [37], let alone attempt to explain how different levels of resources contribute to different levels and combinations of outcomes.

This review highlights the dearth of LHW vaccination strategies that have been evaluated on an economic basis. The very small number of studies identified that evaluated the economic aspects of LHWs promoting or delivering vaccination, as well as the heterogeneity of these studies, makes it difficult to draw conclusions on whether the use of LHWs in vaccination programmes represents good value for the resources invested. The lack of studies is especially surprising given that vaccination is one of the most cost-effective public health interventions [52] and that vaccination comprises a basic component of primary health care and comprises a key part of Millennium Development Goal 4 [53].

It is conceivable that with a larger number of economic evaluations than these three studies, specific characteristics of LHWs in vaccination programmes that could be generalized to help inform decision making would have been identified. The current lack of standardization in the design, analysis and reporting of results from economic evaluations, and substantially different outcomes [54], also lead to a lack of comparability. In this review, outcomes of the included studies were: cost per discounted year of life [17], cost per fully vaccinated child [18] and cost per quality adjusted life year [19]. Though there is a role for peer review to play in upholding and regulating reporting standards for the economic evaluations published [55], as well as in the quality of the studies published, there is also a need for more consistency in adhering to the numerous recommendations and guidelines for conducting economic evaluations [16,56]. This, in turn, would aid the potential of systematic reviews to provide insights for planning and decision making.

Further research on the costs and cost-effectiveness of LHWs in delivering and promoting vaccinations is needed (Table 3), especially with closer examination of: the links between LHW-roles and strengthened primary-care facilities and first-referral services [3]; potential LHW involvement in long-term human resource planning; better training and supportive supervision [57]; the substitution of nursing and other professional tasks by lay workers (e.g. CHWs, pharmacy assistants) [3]; and cost-effective approaches to determining the allocation of PHC services based on health needs [3]. Concomitant with technological advancements in improving the safety, efficiency and thermostability of vaccinations, assessments should be conducted as to how LHWs may provide an increasingly important role in vaccine delivery at the community level.

**Table 3:** Recommendations for future research

To provide decision makers with adequate and useful data on the cost effectiveness of lay health worker interventions for vaccination, future evaluations of such programmes should:	
Compare the costs of alternative options	• include a comparative analysis of costs and consequences of alternative courses of action, or at least a detailed costing of personnel and other resources associated with the intervention

Standardize design, analysis and reporting	• address the current lack of standardization in the design, analysis and reporting of economic evaluations results; in the range of outcomes used; and in the reporting of contextual factors, to improve the comparability of these evaluations

Examine the variability of interventions	• look explicitly at variability between interventions implemented in different locations (within or between countries) and explore how different levels of resources contribute to different levels and combinations of outcomes

Explore types and levels of remuneration	• explore how different levels and methods of remuneration, and types of financial or non-financial incentives, impact on the cost-effectiveness and sustainability of programmes

Vary the evaluation time frame	• explore the impacts on cost-effectiveness of incorporating a longer evaluation time-frame

Capture the instrumental value of LHWs to the communities in which they work*	• assess the impact on cost-effectiveness of using an institutional economics framework, such addressing issues of implicit contracts and informational asymmetries; taking into account governance issues and institutional evolution and transition; and conducting a transaction cost analysis •develop approaches to account for volunteer labour in these programmes

* Jan S, Pronyk P, Kim J: **Accounting for institutional change in health economic evaluation: a program to tackle HIV/AIDS and gender violence in Southern Africa**. *Soc Sci Med *2008, **66**:922-932.

Building on these recommendations, it is proposed that this area of research would also benefit strongly from a randomized community trial, or series of trials, comparing the cost-effectiveness of LHWs for vaccination in a range of low-, middle- and high-income settings. It is important to adhere to existing guidelines for the conduct of cost-effectiveness studies and to build on these by using the holistic economic evaluation framework proposed by Jan et al. [11]. This would aid in incorporating aspects of institutionalist economics, which takes into account context-specific norms and values, and better reflects the wider social value of health programmes within a community. Further to this, taking into consideration sustainability issues will help ensure continuing programme responsiveness to community needs, and allow LHWs to maximise their effectiveness in the context in which they are working.

## Competing interests

The authors declare that they have no competing interests.

## Authors' contributions

SL, IS and CG conceived of the study. AC and DW designed the study, conducted the search and analysis, interpreted the data, and drafted the manuscript. All authors read and approved the final manuscript.

## Appendix 1 - Criteria for evaluation

- Was the perspective from which the costs were measured explicitly stated?

- Were all the important and relevant inputs identified and valued given the viewpoint?

- Were sources of data clearly identified? (list sources)

- Were the unit costs of inputs and quantity clearly identified?

- Was it clear how costs were valued?

- Is there an attempt to calculate economic costs?

- Were base year, details about currency conversion and any adjustment for inflation given?

- Was discounting performed? If yes, was an appropriate justification of the rate given?

- Was sensitivity analysis performed? If yes, were justifications for the choice of variable and their level given?

- Were issues of affordability and/or sustainability discussed?

- Was generalizability discussed by the authors?

- Were transaction costs, or transaction cost savings, estimated?

- Were community norms or values discussed in the context of the institutionalization of LHW programmes?

## Supplementary Material

Additional file 1 **Medline search strategy**. The data provided represent the search terms used in searching for studies in the Medline database. Click here for file

Additional file 2 **Background characteristics of additional costing studies**. The data provided represent in tabular form the background characteristics, such as area studied and vaccines used, of studies using LHWs for vaccine delivery and including some costs, but not meeting the criteria of cost-effectiveness analyses. Click here for file

Additional file 3 **Brief descriptions of included cost studies**. The data provided represent brief descriptions of the costing studies which did not meet the criteria for inclusion as cost-effectiveness studies. Click here for file
