# Supplementary material for: Are vaccination programmes delivered by lay health workers cost-effective? A systematic review
Source: Hum Resour Health. 2009 Nov 3;7:81. doi: 10.1186/1478-4491-7-81 (PMC2780975; doi:10.1186/1478-4491-7-81)
Supplement: Additional file 1 — Medline search strategy. The data provided represent the search terms used in searching for studies in the Medline database. [file 1478-4491-7-81-S1.doc]

# Additional file 1 – Medline search strategy

**MEDLINE Ovid**

1. Community Health Aides/

2. Allied Health Personnel/

3. Home Health Aides/

4. Voluntary Workers/

5. Home Nursing/

6. Community Networks/

7. Peer Group/

8. Caregivers/

9. Social Support/

10. ((lay or voluntary or volunteer? or untrained or unlicensed or nonprofessional? or non professional?) adj5 (worker? or visitor? or attendant? or aid or aides or support$ or personnel or helper? or carer? or caregiver? or care giver? or consultant? or assistant? or staff or visit$ or midwife or midwives)).tw.

11. lay volunteer?.tw.

12. paraprofessional?.tw.

13. (paramedical adj (person$ or staff or aid or aides or assistant?)).tw.

14. (trained adj3 (volunteer? or lay person$ or health worker? or mother?)).tw.

15. ((community or primary or village?) adj3 (health worker? or health care worker? or healthcare worker?)).tw.

16. (community adj3 (volunteer? or aid or aides or support)).tw.

17. ((birth or childbirth or child birth or labor or labour) adj (attendant? or assistant?)).tw.

18. (doula? or douladural?).tw.

19. monitrice?.tw.

20. (peer adj (volunteer? or counsel$ or outreach or support)).tw.

21. "peer to peer".tw.

22. "mother to mother".tw.

23. "family to family".tw.

24. (church based adj3 (intervention$ or program$ or counsel$)).tw.

25. (linkworker? or link worker?).tw.

26. barefoot doctor?.tw.

27. (home adj (care or aid or aides or nursing or support or intervention? or treatment? or visit$)).tw.

28. ((care or aid or aides or nursing or support or intervention? or treatment? or visit$) adj3 (lay or volunteer? or voluntary)).tw.

29. 27 and 28

30. or/1-26,29

31. exp Vaccines/

32. exp Immunization/

33. Immunization Programs/

34. immunizat$.hw.

35. vaccinat$.hw.

36. (vaccin$ or immun$ or inoculat$ or innoculat$).tw.

37. or/31-36

38. 30 and 37

39. Economics/

40. "Costs and Cost Analysis"/

41. "Cost Allocation"/

42. Cost-Benefit Analysis/

43. "Cost Control"/

44. "Cost Savings"/

45. "Cost of Illness"/

46. "Cost Sharing"/

47. "Deductibles and Coinsurance"/

48. Medical Savings Accounts/

49. Health Care Costs/

50. Direct Service Costs/

51. Drug Costs/

52. Employer Health Costs/

53. Hospital Costs/

54. Health Expenditures/

55. Capital Expenditures/

56. Value of Life/

57. Economics, Dental/

58. Economics, Hospital/

59. Hospital Charges/

60. Economics, Medical/

61. Economics, Nursing/

62. Economics, Pharmaceutical/

63. or/39-62

64. (econom$ or cost or costs or costly or costing or price or prices or pricing or pharmacoeconomic$).ti,ab.

65. (expenditure$ not energy).ti,ab.

66. (value adj2 money).ti,ab.

67. budget$.ti,ab.

68. or/64-67

69. 63 or 68

70. letter.pt.

71. editorial.pt.

72. historical-article.pt.

73. or/70-72

74. 69 not 73

75. Animals/

76. Humans/

77. 75 not (75 and 76)

78. 74 not 77

79. (metabolic adj cost).ti,ab.

80. ((energy or oxygen) adj cost).ti,ab.

81. 78 not (79 or 80)

82. 38 and 81
