# Supplementary material for: Are vaccination programmes delivered by lay health workers cost-effective? A systematic review
Source: Hum Resour Health. 2009 Nov 3;7:81. doi: 10.1186/1478-4491-7-81 (PMC2780975; doi:10.1186/1478-4491-7-81)
Supplement: Additional file 2 — Background characteristics of additional costing studies. The data provided represent in tabular form the background characteristics, such as area studied and vaccines used, of studies using LHWs for vaccine delivery and including some costs, but not meeting the criteria of cost-effectiveness analyses. [file 1478-4491-7-81-S2.doc]

# Additional file 2 - Background characteristics of additional costing studies

|  | Barzgar et al. [20] | Berggren [21] | Calderon-Ortiz et al. [29] | Creese [24] | Cutts et al. [25] | Khan et al. [22] | Levin CE et al. [23] | Linkins et al. [30] | Paxman et al. [26] | Rask et al. [27] | Tulchinsky et al. [28] |
| --- | --- | --- | --- | --- | --- | --- | --- | --- | --- | --- | --- |
| Area studied | Pakistan (districts of Chakwal, Malir and Mastung in the provinces of Punjab, Sindh and Balochistan) | Deschapelles, Haiti | Tixtla, Guerrero, Mexico | 16 municipalities in Brazil | Mozambique | Dhaka, Bangladesh | Indonesia (Provinces of East Java, West Nusa Tenggara, and Yogyakarta) | One urban and one rural area of Egypt | Slums of Kolkata, Punjab and Himachal Pradesh, Uttaranachal, India | Atlanta, Georgia, USA | Hebron district, West Bank and Gaza Strip |
| Timing of the study | 1994-1995 | 1968-1972 | April-Dec. 1994 | Jan. – June 1982 | 1986 | 1996 | Aug 2000 –July 2001 | 1993 | 1999-2003 | Sept. 1, 1996 – Mar. 31, 1998 | 1985-1996 |
| Type of intervention | Training of a new cadre of health worker to deliver vaccinations and PHC | Delivery of vaccination via clinics in major outdoor markets | Promotion and delivery of vaccination | Routine, intensified, and campaign delivery of vaccines | Door-to-door vaccine promotion | Vaccine promotion and delivery | Home vs. facility vaccination delivery | Campaign; promotion and delivery of vaccines | NGO service provision for vaccine delivery | Immunisation Registry to promote vaccination uptake | Promotion of PHC and vaccines |
| Type of LHW | Female health workers | Literate lay persons recruited on a day-labour basis | CHWs* | Workers from schools, and private and voluntary organizations | Community representatives; lay individuals from workplaces and schools | EPI Vaccinator at NGO and Govt of Bangladesh static and outreach sites | Midwives | Community workers | Community health volunteers | Children were randomized to one of four study arms, two of which included outreach worker[[1]](#footnote-2) (LHW) phone call follow-up and computer-generated telephone messages with outreach worker backup. | Village health guide/provider of primary health care to villages |
| Training | Basic training over 3 months and for one week a month for a year after, including community organization, health education and promotion and vaccination of infants against six diseases. | On the job, taught and supervised by full-time employees of hospital community health department | Unknown | Unknown | Training for mobilization | EPI- and recurrent training | Already trained | Unknown/ Not stated | Two-weeks, with additional subsequent training (not defined) | Unknown | EPI- and recurrent training |
| Comparator(s) | (Implicitly) Usual care | Usual care | Centrally planned strategy of temporarily contracting of individuals to catch-up vaccination schemes, as needed (not regularly scheduled) | Compared each strategy to each other | Usual care | NGO-run and Government of Bangladesh-run static and outreach facilities. | 10-dose vial of Hep B with disposable syringe administered at health facilities (usual care) | House visit vs. fixed-site polio vaccine delivery | Usual care | Usual care (doing nothing beyond normal clinical procedure) | None |
| Study type | Cost analysis | Cost analysis | Comparison | Comparison | Cost analysis | Evaluation | Cost analysis | Cost comparison | Cost analysis | Cost comparison | Evaluation |
| Vaccines delivered | Six childhood immunisations | Neonatal tetanus | Routine childhood vaccines | Oral polio, measles, DTP, BCG | BCG, DTP, OPV, measles, TT | Routine childhood immunisations | Hepatitis B birth dose | Oral polio vaccine | Routine childhood immunizations | Routine childhood vaccines | Routine childhood immunisations |
| Age group(s) targeted | Not stated | Neonates | Children <1 yr of age | Children of all ages | Children 12-23 months and mothers | Children | Neonates (administered within the first 7 days of birth) | Children 0-5 years old | Not stated | Children <1 yr of age | Children and mothers especially |
| Perspective | Not stated | Hospital (inferred) | Healthcare system (inferred) | Healthcare system (inferred) | Healthcare system (inferred) | Healthcare providers (stated) | Health system | Government health system | NGO providing services | Unknown | Healthcare system (inferred) |
| Study outcome and cost results | Capital cost of recruitments, training and deployment of each female health worker was US$ 386 (1994); inputs for salaries, essential drugs, supervision and other recurrent costs were estimated to be $1.13 per female health worker. | Authors assume 2320 cases of tetanus were avoided, resulting in 41 140 days of hospital care averted, which at US$12 allows re-distribution of US$494 000. Ratio of control cost to cost of no control was 1:7 (dollar value of treatment costs averted was more than 7 times the cost of the programme). | Intervention strategy of using 2 people known to the community 1-2 times a week to cover an area containing 100 children for US$ 12.70 per person per week versus as needed, resulted in only 42 days needed to cover 100 children vs the control group which needed 60 days, resulting in a cost of US$533.43 (intervention) vs US$762.05. | Cost per fully immunized child ($US):  Polio: 2.04 (campaign) – 4.77 (routine)  Measles: 1.74 (intensification) – 4.63 (routine) | Cost per fully vaccinated child: $6.90 | NGO static facilities were more cost-effective (US$ 11.50) than the government static facilities (US$ 12.93). The NGO outreach sites were also more cost-effective than the government outreach sites (US$ 9.80 and US$ 10.07 per MVC respectively). | The use of village  midwives to administer a birth dose using the Uniject device  during a home visit is cost-saving when the wastage rates of the multi-dose vial alternative are more than 33%. | Cost per child vaccinated (US$):  Fixed-site delivery urban; House-to-house delivery urban and rural: 0.11  Fixed-site delivery rural: 0.14  Cost per high-risk child vaccinated (0-1 OPV doses received) (US$):  Fixed-site delivery urban: 3.20  Fixed-site delivery rural: 6.31  House-to-house delivery urban: 1.46  House-to-house delivery rural: 1.51 | Average cost per service of $3.11 (service includes child immunization, family planning, safe motherhood and limited curative care). | Outreach (registry based): $1.87  Autodialer (registry based): $1.34  Combination (registry based): $2.76 | On average, each VHR costs $3.66 per villager in one-time start-up costs, and $6.18 per villager per year to operate. This covers all care that is provided free-of-charge and includes vaccines, iron, vitamins, ORS, blood tests and screening tests (for PKU and hypothyroidism). |
| Evaluation funded by | Not stated | Grant Foundation of Pittsburgh, Pennsylvania | NA | Not stated | Not stated | Department for International Development (DfID), UK | Children’s Vaccine  Program of the Program for Appropriate Technology in Health  (PATH) | Not stated | Not stated | CDC | Terre des Hommes, Israeli Cancer Society, UNICEF, WHO |

* Two locally recruited known and knowledgeable residents, compensated at US$12.70 a day, employed for 1-2 days a week. For the purpose of this review, Mexican pesos (N$)were converted to US$ based on the exchange rate at the mid-point of the study period, August 1, 1994 (N$ 1 = US$ 0.294).

1. The outreach worker is the lay health worker in this instance. [↑](#footnote-ref-2)
