# Supplementary material for: Are vaccination programmes delivered by lay health workers cost-effective? A systematic review
Source: Hum Resour Health. 2009 Nov 3;7:81. doi: 10.1186/1478-4491-7-81 (PMC2780975; doi:10.1186/1478-4491-7-81)
Supplement: Additional file 3 — Brief descriptions of included cost studies. The data provided represent brief descriptions of the costing studies which did not meet the criteria for inclusion as cost-effectiveness studies. [file 1478-4491-7-81-S3.doc]

# Additional file 3 – Brief descriptions of included cost studies

Barzgar et al. [20] reported on the effects of female health workers residing in villages in Pakistan and who were recruited and trained to deliver primary care, including delivering vaccinations from 1994-1995. Capital cost of recruitments, training and deployment of each female health worker was $386 (1994); inputs for salaries, essential drugs, supervision and other recurrent costs were estimated to be $1.13 per female health worker [20].

Berggren [21] reported on the role of auxiliary health workers in controlling tetanus in a rural area in Haiti through vaccine delivery. The author estimated that, based on the results of the vaccination programme, 41,140 days of hospital care for neonatal tetanus were averted. At a cost of $12 per hospital day this resulted in $494,000 effectively redistributed to the care of other diseases [21].

Calderon-Ortiz and Mejia-Mejia [29] evaluated the results of a new vaccination strategy for children less than one year old, involving hiring local community individuals in an urban area of Tixtla, Mexico, compared to a control group which had periodic vaccination campaigns. Individuals from the community were hired to promote and vaccinate for the early programme recruitment of children less than two months old and for the vaccination of children with incomplete vaccination schemes. Results showed that in the intervention area, vaccinators required 42 days to vaccinate approximately 100 children at a total cost of US$ 533.43, while it took 60 days to vaccinate approximately 100 children in the control area for a cost of US$ 762.05. For the purpose of this review, Mexican pesos (N$) were converted to US$ based on the exchange rate at the mid-point of the study period, August 1, 1994 (N$1 = US$ 0.294).

Creese [24] reported costs for routine childhood immunization programmes delivering polio and measles vaccination, for children of all ages, by LHWs, which included workers from schools and private and voluntary organisations in 16 Brazilian municipalities between January and June 1982. Creese found that in the case of vaccination with oral poliovirus vaccine, average costs per dose fall from ~$1.59 to $0.68 when routine and mass campaign strategies are compared [24]. Costs per fully immunized child for polio ranged from $2.04 (campaign) to $4.77 (routine), and for measles, costs per fully vaccinated child were between $1.74 (intensification) and $4.63 (routine) [24].

In Mozambique in 1986, Cutts et al. [25] showed increased vaccination coverage of routine childhood vaccinations amongst children 12-23 months in a door-to-door canvassing strategy involving local community representatives and lay individuals from workplaces and schools, as well as volunteers from local organisations to promote vaccination. Immunisation costs were estimated to be $6.90 per fully vaccinated child [25].

Khan et al. [22] estimated the costs of providing child immunisation services in Dhaka, Bangladesh based on facility-level surveys of vaccinators delivering vaccines in 1999. If all immunisation doses were administered correctly, a fully-vaccinated child would incur an average hypothetical cost per child of $6.91. Rather, authors found that the average cost per measles vaccinated child (MVC, a proxy measure for ‘fully vaccinated child’) was $11.61, indicating significant partial vaccinations or double-dosing. The authors found that cost per MVC was the highest ($12.93) for the government static sites and was the lowest ($9.80) for the NGO outreach sites, as the NGO-run sites usually employed only vaccinators, whereas the government sites also employed physicians, which drove costs up.

Levin et al. [23] presented a retrospective cost analysis of village midwives in Indonesia delivering a birth dose of hepatitis B vaccine administered to infants within seven days of birth, using the Uniject pre-fill injection device, between August 2000 and July 2001. The authors found that the introduction of hepatitis B vaccine prefilled in Uniject single-dose injection devices for use by midwives for delivering the birth dose is cost-saving compared to usual care, when the wastage rate for multi-dose vials is greater than 33% [23].

Linkins et al. [30] compared per child vaccination costs for house-to-house versus fixed-site oral poliovirus vaccine (OPV) delivery in Egypt in 1993, using community health workers alongside health personnel such as doctors and nurses, to vaccinate. In urban areas, the cost per child vaccinated was similar for both fixed-site and house-to-house vaccinations ($0.11). In rural areas, it was higher for fixed-site delivery than for house-to-house delivery ($0.14 vs. $0.11) [30].

Paxman et al. [26] reviewed the India Local Initiatives Program (India-LIP) where, from 1999 to 2003, three Indian nongovernmental organizations (NGOs), often using community health volunteers, provided services such as routine childhood vaccinations, for 784,000 people in the slums of Kolkata and the states of Punjab, Himachal Pradesh and Uttaranachal. Community resources, such as local health personnel, community-supplied clinic sites, and community drug funds, added 40 cents to every dollar provided by donors, while average cost per service was $3.11 (‘service’ includes child immunization, family planning, safe motherhood and limited curative care) [26].

Rask et al. [27] prospectively measured the cost of three equally effective registry-based interventions used to promote childhood vaccination in the United States from September 1996 to March 1998, evaluated how the size of the targeted population affects cost estimates, and compared these results with previously reported studies. A total of 3050 children aged less than 12 months were randomized to one of four study arms (including usual care), with monthly costs of the three registry-based intervention types being (1) autodialer (automated telephone call), $1.34 per child; (2) outreach worker, $1.87 per child, and (3) combination, $2.76 per child.

Tulchinsky et al. [28] evaluated the Village Health Room (VHR) programme established in villages with no on-site health facilities in the Hebron District of the West Bank and Gaza Strip, from 1985 until 1996. Each VHR is staffed by a Village Health Guide selected from female village residents in their early 20s who had completed a minimum of 10-11 years of education and would work in their own villages after training for 6-8 months. It was estimated that on average each VHR costs $3.66 per villager in one-time start-up costs and $6.18 per villager per year to operate, which included delivering vaccines as well as vitamins and oral rehydration solution (ORS).
